# Differences in microbiome and virome between cattle and horses in the same farm

**DOI:** 10.5713/ajas.19.0267

**Published:** 2019-10-22

**Authors:** Jongbin Park, Eun Bae Kim

**Affiliations:** 1 Department of Animal Life Science, College of Animal Life Science, Kangwon National University, Chuncheon 24341, Korea; 2 Department of Applied Animal Science, College of Animal Life Science, Kangwon National University, Chuncheon 24341, Korea

**Keywords:** Horse, Cattle, Virome, Microbiome, Environment

## Abstract

**Objective:**

The ecosystem of an animal farm is composed of various elements, such as animals, farmers, plants, feed, soil, and microorganisms. A domesticated animal’s health is largely connected with the reservoir of bacteria and viruses in animal farms. Although a few studies have focused on exploring the gut microbiome of animals, communities of microbiota and viruses in feedlots have not been thoroughly investigated.

**Methods:**

Here, we collected feces and dust samples (4 groups: cattle feces, C_F; horse feces, H_F; cattle dust, C_D; and horse dust, H_D) from cattle and horse farms sharing the same housing and investigated their microbiome/virome communities by Illumina sequencing.

**Results:**

Dust groups (C_D and H_D) showed higher microbial diversity than feces groups (C_F and H_F) regardless of animal species. From the microbial community analysis, all the samples from the four groups have major phyla such as Proteobacteria (min 37.1% to max 42.8%), Firmicutes (19.1% to 24.9%), Bacteroidetes (10.6% to 22.1%), and Actinobacteria (6.1% to 20.5%). The abundance of Streptococcus, which commonly recognized as equine pathogens, was significantly higher in the horse group (H_D and H_F). Over 99% among the classified virome reads were classified as Caudovirales, a group of tailed bacteriophages, in all four groups. Foot-and-mouth disease virus and equine adenovirus, which cause deadly diseases in cattle and horse, respectively, were not detected.

**Conclusion:**

Our results will provide baseline information to understand different gut and environmental microbial ecology between two livestock species.

## INTRODUCTION

The environment in the feedlot, which is the animals’ living area, was constructed by diverse environmental factors [1]. Farmers and animals are the main contributors which make the feedlot environment. Inorganic environmental factors such as dust, feed, climate, fertilizers, and soil also make farm environment. These complex compositions produce millions of diverse bacterial/viral reservoirs that influence animal health, diseases, performance, and immunities [2,3]. Foot-and-mouth disease (FMD), one of the most critical viruses that affects cloven-hoofed animals, can survive in the harsh condition not only in the host animals, but also in manure, soil, feed, and agricultural devices for extended periods of time [4]. Likewise, a number of health-associated diseases are endemic in animal farms and infect both farmers and animals. However, only a limited number of studies have explored the relationships between the animal health and feedlot microbial communities.

Both cattle and horses represent herbivores among ruminants and non-ruminants, respectively. Development of next-generation sequencing technology has enabled the analysis of gut microbiomes of animals. The gut microbiome in both animals has been well studied, because of their importance in the livestock industry [5]. This approach allows the understanding of host-gut microbiota interactions. Previous studies have revealed the microbial abundance and diversity of animals during different growth stages, feed consumptions, and environments [6,7]. Recent studies have focused on the airborne bacteria from feedlots, and found that it can affect the farmer’s or animal’s nasal bacterial communities and their health [8]. In cattle, feedlot bacterial communities are more diverse than flora, and can spread many airborne bacterial diseases, like bronchopneumonia [9]. In horses, airborne components like pollen, dust, and bacteria can cause asthma-like diseases [10]; however, currently there are only a limited number of examples. Thus, further studies are required to improve our understanding of the ecology of feedlot microbes. The aim of this study is to characterize the microbial/viral communities and investigate the host/environment effects of microbial/viral communities in feedlots. To do this, we compared microbial/viral diversity in cattle and horse which share their habitat.

## MATERIALS AND METHODS

### Sample collection and ethics approval

**Figure 1:**
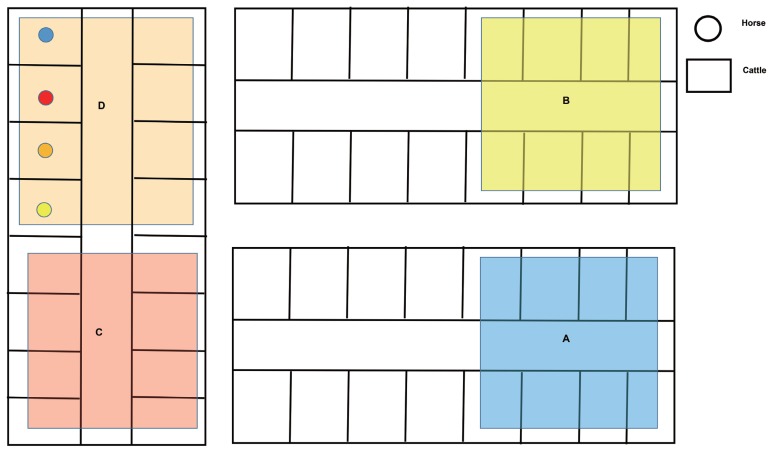
Information of sampling sites. Alpabet means sampling site: A, cattle fattening; B, cattle_weanling; C, cattle dairy; D, horse_fattening.

Cattle and horse samples were obtained from a Kangwon National University experimental farm (Chuncheon, Korea). All feedlots were steel roofed without side wall paved with concrete and bedded with sawdust and soil. Of a total of three cattle feedlots, two feedlots (A, B) were breeding only cattle and one feedlot was breeding both cattle and horses (C and D, Table 1, Figure 1). Feedlot A raised 21 Hanwoo, feedlot B-1 raised 11 Hanwoo, feedlot B-2 raised 6 Holsteins, feedlot C and D raised 33 Hanwoo and 4 horses, respectively (Supplementary Table S1). Animals in all areas have different birth of date and sex. Only horses grazed over once a week near the feedlot. Cattle and horses provided straw freely and feeding commercial feed from a local company (Easyfarms, Cheonan, Korea) during the experiment period in Table 2. We randomly collected 5 fresh feces and 5 dust/soil mixture than pooled in one tube in each feedlot. Totally, five feces and 4 dust from 4 cattle feedlot and 4 feces and 4 dust from horses feedlot were analyzed in this study (cattle feces, C_F; horse feces; H_F, cattle dust; C_D, horse dust; H_D). All experimental procedures were performed in accordance with the Guide for the Care and Use of Laboratory Animals and approved by the Institutional Animal Care and Use Committee of Kangwon National University (KIACUC, KW-161101-1).

### DNA extraction and sequencing for microbiome

DNA was extracted from 5 g of each fecal and dust-soil mixture sample using a NucleoSpin Soil kit (Macherey-Nagel, Düren, Germany). Briefly, each samples were put into the NucleoSpin bead tubes containing 0.6 to 0.8 mm ceramic beads and homogenized using the taco Prep bead beater (GeneReseach Biotechnology Corp., Taichung, Taiwan). Next step was followed according to the manufacturer’s recommendations and extracted genomic DNA was stored at −20°C until further analysis. The 16S ribosomal RNA V4 region gene was amplified from the total extracted genomic DNA using Takara Ex-taq DNA polymerase (Takara Bio, Shiga, Japan) and universal primer sets (Forward: 5′-GGACTACHVG GGTWTCTAAT-3′ and R: 5′-GTGCCAGCMGCCGC GGTA A-3′) [6]. Before sequencing, amplified genomic DNA was normalized to 50 ng per sample using Spark 10M multimode microplate reader (Tecan Group AG, Zurich, Switzerland). For Illumina sequencing, a DNA library was constructed and sequenced by C&K Genomics. The DNA libraries were sequenced in the Illumina MiSeq platform (Illumina Inc., San Diego, CA, USA) generating 2×250 bp paired-end.

### DNA/RNA extraction and sequencing for virome

To analyze total nucleic acid viruses in the samples, 5 g of feces and dust-soil were mixed with 10 mL of ultra-distilled water. Mixed samples were filtered with 100-μm pore-size cell strainer (SPL Life Sciences, Pochen, Korea) and then filtered twice using a 0.2-μm pore-size Minisart syringe filter (Satorious AG, Göttingen, Germany). A total of 200 μL of mixture was used for viral DNA/RNA extraction using an Allprep PowerViral DNA/RNA Kit (Qiagen, Valencia, CA, USA), according to the manufacturer’s protocol. Extracted DNA/RNA mixture was prepared cDNA and amplified using a QuantiTect Whole Transcriptome kit (Qiagen, USA). Briefly, RNA was synthesized and ligated to cDNA using T-Script enzyme and reverse transcriptase. Then, cDNA and DNA mixture was amplified using randomly amplified using Phi 29 polymerase (Qiagen, USA). The cDNA and DNA libraries were constructed (C&K Genomics, Seoul, Korea) using Nextera XT sample prep kit (Illumina Inc., USA) and sequenced (Macrogen, Seoul, Korea) in the Illumina HiSeq X platform (Illumina Inc., USA).

### Bioinformatics for microbiome

The raw sequence reads were quality-trimmed and de-multiplexed. Processed reads were analyzed using open-source bioinformatics pipeline for microbiome, quantitative insights into microbial ecology (QIIME, http://qiime.org/index.html) version 1.9.1 software to analyze microbial community richness and diversity indices (rarefaction curves, chao1, and shannon). Reads were clustered into operational taxonomic units (OTUs) by nominated close-reference OTU picking at 97% identity with the GreenGenes 16S rRNA sequence database (ver. 13–8, http://greengenes.secondgenome.com/) as the references. OTU tables was normalized to 15,000 reads per sample by single rarefaction. Beta diversity principal coordinate analysis (PCoA) was performed based on UniFrac distances and visualized with EMPeror 3D visualization software. To predict functional and evolutional genes from the microbiota, biological observation matrix file including information of OTUs generated by QIIME was compared to the database of clusters of orthologous groups of proteins (COGs, https://www.ncbi.nlm.nih.gov/COG/) and Kyoto encyclopedia of genes and genome (KEGG, https://www.genome.jp/kegg/) pathways. Phylogenetic Investigation of Communities by Reconstruction of Unobserved States (PICRUSt), designed to predict metagenome functional content from genes, was used for the prediction of KEGG and COGs using normalized OTUs (http://picrust.github.io/picrust/). Finally, we performed a linear discriminant analysis (LDA) effect size (LEfSe) analysis for statistical significance, features of each sample, and visualization. We put into microbial abundance tables in the web-based platform Galaxy (https://huttenhower.sph.harvard.edu/galaxy/) for LEfSe analysis. Statistical analysis and visualization were performed using the R statistical package version 3.5.0 (R Foundation for Statistical Computing, Vienna, Austria).

### Bioinformatics for virome

Whole genome sequencing reads were quality trimmed using an in-house Perl script and Cutadapt 1.14 (https://cutadapt.readthedocs.io/en/stable/#). Processed reads were taxonomically classified with the pre-built 8 GB database constructed from complete bacterial, archaeal, and viral genomes in RefSeq (Oct. 18, 2017) using the Kraken (Version 1, https://ccb.jhu.edu/software/kraken/) algorithm-based taxonomic sequence classification system with kraken −db refseq, --threads 24, and --paired options. Classified reads were collected and counted using an in-house Perl script.

## RESULTS

### Microbial community and diversities in different origins

**Figure 2:**
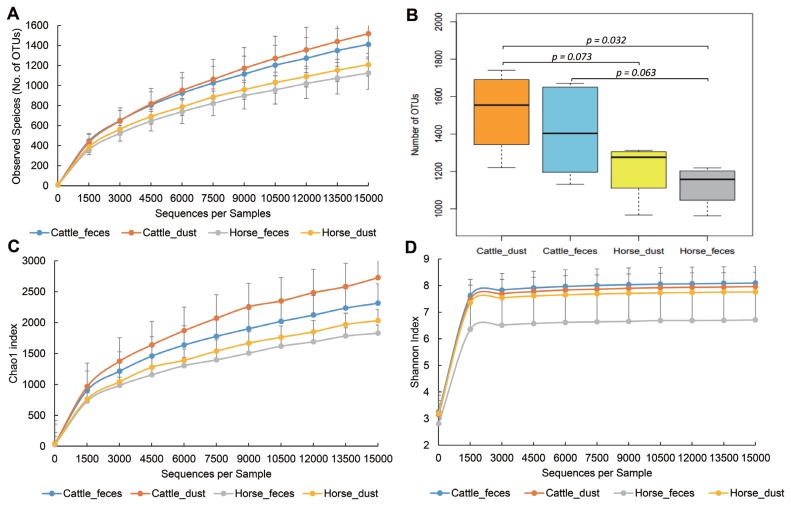
Rarefaction analysis observed species (No. of operational taxonomic units), Chao1 index, and Shannon index obtained from various farm groups. Lines represent the mean and error bars represent standard deviations. Color means: blue, cattle_feces; orange, cattle_dust; silver, horse_feces; yellow, horse_dust.

**Figure 3:**
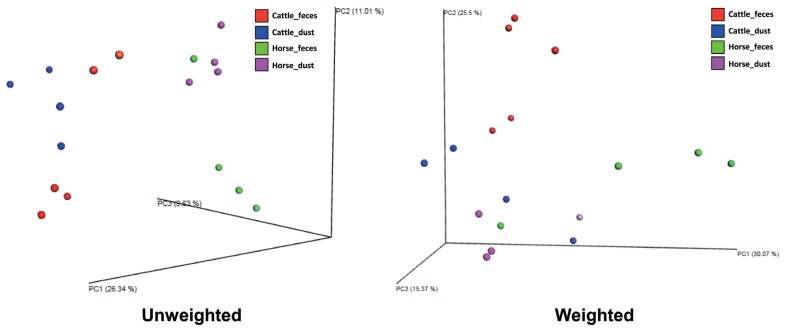
Principal coordinate analysis of unweighted and weighted based on UniFrac distances. Subject color: red, cattle_feces (n = 5); blue, cattle_dust (n = 4); green, horse_feces (n = 4); purple, horse_dust (n = 4).

To compare the bacterial diversity and communities among the groups, OTUs were randomly selected at different reads in each sample (10, 1,509, 3,008, 4,507, 6,006, 7,505, 9,004, 12,002, 13,501, and 15,000) to analyze bacterial diversity and richness (Figure 2). Cattle groups showed a higher number of OTUs than the horse group in both sample types (p<0.1, Figure 2A, 2B). Each dust group showed a higher number of observed species than the feces group. Cattle groups also showed a higher value than horse groups in the Chao1 and Shannon index. PCoA based on UniFrac distances showed the relationships of bacteria diversity among the samples (Figure 3). First, samples were clustered together according to their origin (cattle or horse), and then clustered again by their sample type (feces or dust) in an unweighted level. In the weighted level, C_F group and H_F group were separately clustered again, but between C_D and H_D, several samples were clustering together. There were no differences within the species.

### Relative abundance of bacteria from different samples

**Figure 4:**
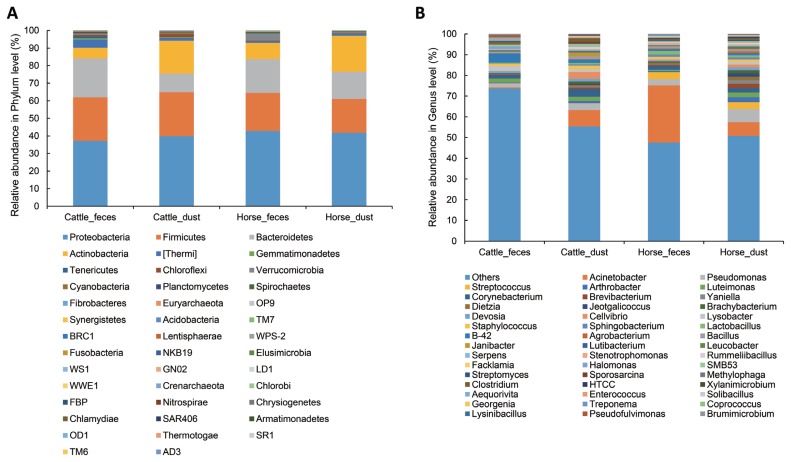
Relative abundance of bacterial community at phylum (A) and genus (B) level in the different origins. Under the 0.05% of relative abundance in genus levels were re-classified to others.

To determine which bacteria taxa determine a separation of the groups, we compared bacteria relative abundance at the phyla and genus level from the four targeted groups; C_F (n = 5), C_D (n = 4), H_F (n = 4), H_D (n = 4). To compare between groups, each group was compared to one other group (C_F vs C_D, H_F vs H_D, C_F vs H_F, and C_D vs H_D), respectively. The most abundant phyla in all groups were Proteobacteria, Firmicutes, Bacteroidetes, and Actinobacteria (Table 3, Figure 4A). In Archaea, only a small portion of Crenarchaeota (min 0.000% to max 0.002%) and Euryarchaeota (min 0.079% to max 0.51%) were detected in all group (Table 3). In the comparison of abundance between C_F and C_D, Verrucomicrobia (paired t-test, p = 0.018), Bacteroidetes (p = 0.009), and Tenericutes (p = 0.016) were significantly higher in C_F. There were no differences in the comparison between H_F and H_D. In the comparison between C_F and H_F, Acidobacteria, Cyanobacteria, Gemmatimonadetes, Chloroflexi, Tenericutes, Thermi, and candidate division NKB19 bacterium are significantly more abundant in C_F (p<0.05).

**Figure 5:**
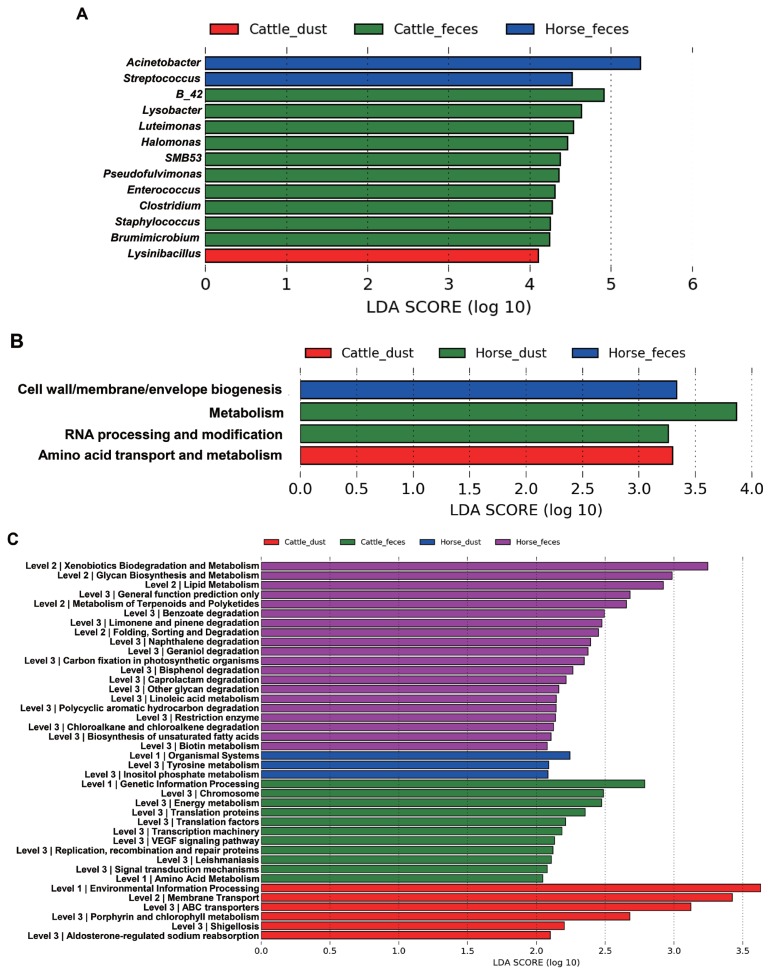
Different composition of microbiota among the samples in genus level and different functions predicted by PICRUSt at the COGs and KEGG pathways. Histogram of genus (A), COGs (B), and KEGG pathway (C) from LEfSe analysis. PICRUSt, Communities by Reconstruction of Unobserved States; COGs, clusters of orthologous groups; KEGG, Kyoto encyclopedia of genes and genome; LEfSe, a linear discriminant analysis effect size.

The relative abundance at the genus level showed a different abundance pattern among groups (Table 4, Figure 4B). B-42 (4.4%) is the predominant genus in C_F, while Acinetobacter is the predominant genus in C_D, H_F, and H_D. Particularly in H_F, the abundance of Acinetobacter is three times higher than the other groups. Through the LEfse analysis, we reveal that several bacteria groups at the genus level were significantly higher in C_F, C_D, and H_F (Figure 5A). In C_D, *Lysinibacillus* is significantly higher than other groups. C_F was significantly higher in B-42, *Lysobacter*, *Luteimonas*, *Halomonas*, candidate division SMB53 bacterium, *Pseudofulvimonas*, *Enterococcus*, *Clostridium*, *Staphylococcus*, and *Brumimicrobium*. *Acinetobacter* and *Streptococcus* were significantly higher in H_F group. We also compared the relative abundance between the groups (Table 5). In the comparison between C_F and C_D, *Halomonas* was significantly higher in C_F, and *Georgenia*, *Bacillus*, and *Staphylococcus* were significantly higher in C_D. In C_F vs H_F, *SMB53*, *Halomonas*, *Clostridium*, *Lysobacter*, B-42, *Brumimicrobium*, and *Luteimonas* were significantly higher in C_F, and only *Streptococcus* was significantly higher in H_F. In C_D vs H_D, *Bacillus* and *Lysinibacillus* were significantly higher in C_D and *Streptococcus* was significantly higher in H_D, followed by HTCC and *Lactobacillus*. Interestingly, *Streptococcus* was significantly higher in both horse groups compared to cattle groups. In H_F vs H_D, only *Luteimonas* was higher in H_D.

### Functional and evolutional prediction of the microbiota from different samples

**Figure 6:**
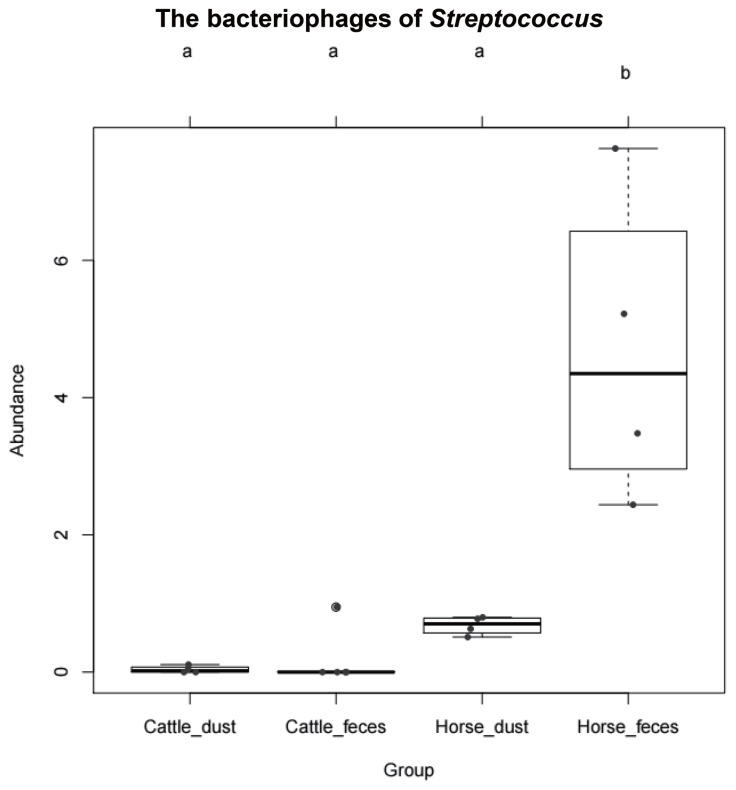
The abundance (%) of *Streptococcus* bacteriophage among the group. Each dot means each sample. b mean difference is significant at 0.05 level.

From the LDA effect size (LEfse), several COGs and KEGG pathway classes were significantly different between the sample types (Figure 6). In COGs, the ‘Cell wall/membrane/envelope biogenesis’ pathway was significantly higher in H_F, ‘metabolism’ and ‘RNA processing and modification’ pathways were significantly higher in H_D, and ‘amino acid transport and metabolism’ pathway was significantly higher in C_D, and significantly different pathways were not found in C_F (Figure 5B). In KEGG, each group showed the significant different in the several pathways and metabolism respectively (Figure 5C). Translation related pathways are significantly higher in C_F, and degradation and metabolism related pathways are significantly higher in H_F.

### Classification of viral sequences

We compared the virus classifications and compositions between the different environments. From each filtered sequencing read, 1.96% to 30.5% of the sequencing reads were classified from the reference database, which include archaea, bacteria, and viruses (Table 6). Among them, most of the reads were classified as bacterial sequences (98.0% to 99.6%). Only 0.12% to 0.48% of sequencing reads were classified as a virus in either collection. Based on the classification of the International Committee on Taxonomy of Viruses (ICTV) and NCBI RefSeq Viral database, we classified each sequence to the Reference database. In the order level, 11.72% to 34.37% reads per group were unclassified. Among the classified reads, *Caudovirales* showed the highest classified ratio (C_F, 64.96%; C_D, 66.84%; H_F, 84.10%; H_D, 87.86%) in all group, followed by *Herpesvirales*, *Bunyavirales*, and *Nidovirales* (Table 7). In the species level of virus classification, major genus groups are gemycircular virus, *Escherichia*, *Pseudomonas*, and *Gordonia* (Table 8). The bacteriophages of *Streptococcus* showed the higher abundance in horse group than cattle group like microbiome results (Figure 6). Deadly disease to cattle and horse, FMD virus and equine adenovirus were not detected.

## DISCUSSION

In this study, we compared the microbial/viral communities between cattle and horses at shared feedlots which had never tried before. The microbiome of herbivore feces are influenced by what they eat, where they live, and by what type of ruminant they are [11]. In microbial diversity, we found that the diversity indices are higher in cattle-related environments in than horse-related environments (p<0.1, Figure 2). Cattle have four stomachs, rumen, reticulum, omasum, and abomasum, whereas horses are monogastric [12,13]. Each stomach has different roles in the cattle. Rumen can digest many kinds of grass through billions of bacteria, protozoa, molds and yeasts. Honeycomb shaped reticulum involved in rumination for better digestion of grass. Omasum filtering large particles and help water resorption. Abomasum is a true stomach producing acid and protease like monogastric animals [13]. Many products such as carbon dioxide, methane, volatile fatty acids, short chain fatty acids (SCFA) were generated in the cattle through digestion of cellulose and hemicellulose [13,14]. These products may be an influential factor in cattle having a much higher bacterial diversity than horses, even though they are both herbivores [14]. Several studies have compared ruminants and monogastric animals, and have found that other ruminants other than cattle (goat, sheep, and deer) have a more diverse microbiome than non-ruminants in general [15]. In bacterial composition, the dominant phyla in both cattle feces and horse feces were Proteobacteria (C_F, 37.15%; H_F, 42.85%), Firmicutes (C_F, 24.82%; H_F, 21.62%), Bacteroidetes (C_F, 22.1%; H_F, 19.19%) and Actinobacteria (C_F, 6.1%; H_F, 9.243%). These results support previous studies that show domestic herbivores share a core fecal microbiota [5,16]. Dust groups also showed a similar pattern with feces group, but the abundance ratio was different. Manure can contaminate fans, water fountains, and barriers because of animal behavior and during cleaning. We guess certain species of bacteria in the contaminate region by feces can survive to activate their survival mechanisms and survive environment like dust. For example, the proportion of gut obligate anaerobic genus *Bifidobacterium* and *Clostridium* showed no significant different between the feces and dust. Certain strains of anaerobic genus *Clostridium* can make endospore to survive in the aerobic condition and certain strains of *Bifidobacterium* acquired tolerance to oxidative stress [17,18]. Likewise, anaerobic microbes can survive in aerobic condition by adaptation using their own defense mechanisms and construct similar pattern. However, more researches need to reveal the survival mechanism of each gut anaerobic bacteria in the environment. Through diversity indices, we revealed dust samples in both groups were more diverse than feces samples. As an example, phylum Actinobacteria showed a higher bacterial composition than feces groups. Actinobacteria have a generalist lifestyle allow them to live in various environments, like plants, gastrointestinal tracts, oceans, and soils [19]. Among them, soils are the major habitat of Actinobacteria. Actinobacteria found in soils have various roles, such as recycling biomaterials, producing bacteriocin, and plant health [20]. Such Actinobacteria groups may influence the nasal or gut bacteria compositions of animals. A total of two archaea phyla, Crenarchaeota and predominant in the cattle, Euryarchaeota was present only a small proportion in all group. We guess bacteria enrichment condition or outer environment was not proper to survive archaea. In Cattle, *Luminococcus*, *Lactobacillus*, *Clostridium*, and *Lysobacter* that have been reported in previous research was also identified in this study [21]. Previous research revealed the predominant bacteria was preserved even under different diet type, continent, and host species [16]. However, dominant in cattle, *Prevotella* was not a major in this study. The relative abundance of *Prevotella* may replace affected by its phylotype or host ages [22]. *Luteimonas*, found in three group (C_F, C_D, and H_D) is frequently found in diverse environment such as soil, wastewater, and ammonia biofilter [23]. Certain genus candidate B-42, *Halomonas*, and *Luteimonas* newly detected in this study. We supposed there are one of distinct microbial features on each farm condition. In horse groups, one of the major genus, *Streptococcus*, was significantly higher in cattle groups (Table 5). *Streptococcus* is one of the major bacteria in the horse gut, together with several Firmicutes groups [24]. Certain strains of *Streptococcus* can help digestion by producing lactic acid [25]. However, several studies reported that many pathogenic diseases, like respiratory and reproductive infection, were caused by *Streptococcus* [26]. Streptococcal infections are a critical issue in the horse industry. We assume that *Streptococcus* was well adapted in the horse farm environment and that it caused an increasing number of streptococcal related infections. Likewise, certain bacteria were influenced by their host, diet, and environment. When they manage to tolerate the ascribed conditions and form communities, they can inversely influence the host, environment, and diet conditions. Through our findings, we revealed several bacteria compositions were influenced by the host, diet, and environment. However, there remains a vast number of bacteria in the gut and environment for which we still do not know their relationships and additional studies are required.

In COGs, bacterial pathways such as metabolism (H_D), RNA processing and metabolism (H_D), amino acid transport and metabolism (C_D), cell wall/membrane/envelope biogenesis pathways (H_F) were present. Despite each pathway have different proportion within groups, all pathways observed in this study are essential for the survival of bacteria [27]. However, we couldn’t reveal why the results have shown different respectively among groups. In KEGG, each group also showed different pathways. Genetic information processing, translation, and transcription metabolism pathways are higher in C_F. Transcription and translation processes are essential for the normal expression of proteins as well as cell survival [27]. These pathways may essential to survive in the cattle gut. In H_F, glycan biosynthesis and metabolism, lipid metabolism, linoleic acid metabolism, biotin metabolism, and biosynthesis of unsaturated fatty acids. Gut microbes in this group may develop these mechanisms to use nutrients or fulfill lack of nutrients in the horses gut. Interestingly, foreign substances degradation pathways such as xenobiotics biodegradation and metabolism, benzoate degradation, naphthalene degradation, limonene and pinene degradation, and bisphenol degradation were significantly higher in H_F but not in C_F. Especially, bisphenol, naphthalene, and benzoate are known as endocrine disruptors that found in pesticide. We guess the development of these microbial pathways in horses are causation after intake grass in the contaminated soil during grazing [28]. However, it needs to reveal the exact differences of metabolisms through further research.

In virus classification, the sequence reads in all four groups were mostly classified to prokaryotic DNA virus *Caudovirales*. In the cattle groups, the most critical virus to cloven-hoofed animals, FMD virus (classified as genus; *Aphthovirus*), was not detected. Horse related eukaryotic virus sequences are also not detected in this study. *Caudovirales* are tailed bacteriophages that are composed of double-stranded DNA (dsDNA), and contain *Myoviridae*, *Podoviridae*, and *Siphoviridae* [29]. The majority of bacteriophages are affiliated to the order Caudovirales. Interestingly, the amount of *Streptococcus* phages was higher in horse group that similar as results of microbiome. We presume that bacteria population influence the bacteriophages population through phage-host interaction. Bacteriophages are known as a key player in the environment because of their ability of bacterial infection and lysis. They use bacteria as a host through infection and they maintained their species via lysogenic and lytic cycle. Several virome studies revealed the main reservoir in the environment like aquatic condition, human feces, wastewater, and fermented foods are prokaryotic viruses [30]. It would be great value to investigate these viruses genetic/biochemical diversities.

In the comparison of whole genome sequencing reads for virome, most of the sequencing reads were classified as bacteria (over 99% in all samples), and only a few reads were classified as viruses and archaea. From these results we identified two possible causes. First, most bacteria or bacterial DNA may not have been filtered during the filtering step. Second, there may be a genome size issue between bacteria and virus. The genome size of bacteria can range from 130 kbp to over 14 Mbp, whereas the largest genome size in viruses is still lower than in bacteria. Sequencing to classify microbiomes or viromes from metagenome samples does not consider the individual organism’s genome size. When the genome size is large, more amplification and sequencing outputs are obtained than in small genomes. Our findings extend the understanding of microbial and viral ecology of cattle and horse environments, and provide a new insight for further study.

## Supplementary Data



## Figures and Tables

**Table 1:** Information of sampling sites

No.	Group	Feedlot	Location	Population	Species	Sample type
1	Cattle	Fattening	A	10 weaning, 10 fattening, 1 Suckling	Hanwoo	Feces
2						Soil and dust
3		Weaning	B-1	7 weaning, 3 breeding, 1 suckling	Hanwoo	Feces
4						Soil and dust
5		Dairy	B-2	6 dairy (3 pregnant, 3 breeding)	Holestein	Feces
6						Soil and dust
7		Breeding	C	13 breeding, 11 pregnant, 3 weaning, 4 suckling, 2 fattening	Hanwoo	Feces
8						Soil and dust
9		Weanling	C		Hanwoo	Feces
10	Horse	Fattening	D_Yellow*	1 fattening	Halla	Feces
11						Soil and dust
12			D_Orange	1 weaning	Halla	Feces
13						Soil and dust
14			D_Red	1 pregnant	Halla	Feces
15						Soil and dust
16			D_Blue	1 fattening	Throughbred	Feces
17						Soil and dust

* See Figure 1(D) for different sampling sites (color).

**Table 2:** Ingredients and chemical composition of diets during an experimental period

Items	Diet				

	Cattle				Horse

	Fattening	Weaning	Dairy	Breeding/pregnant	
Ingredients					
Corn	11)	1	1	1	1
Brans	1	1	1	1	0
Wheat	1	1	1	1	0
Rye	1	0	0	0	1
Oats	0	0	0	0	1
Alfalfa	0	0	1	0	0
Cassava	1	1	1	1	0
Soybean	1	1	1	1	1
Vitamin complex	1	1	1	1	1
Limestone	1	1	1	1	1
Salt	1	1	1	1	1
Molasses	1	1	1	1	1
Minerals (Zn, Mn, Fe)	1	1	1	1	1
Lecithin	1	1	1	0	0
Sodium bicarbonate	1	0	0	0	0
Saccharin	1	0	0	0	0
Calcium phosphate	0	0	0	0	1
porphyry	1	1	0	0	0
Probiotics	0	1	0	0	0
L-carnitine	0	0	0	0	1
Tocopherol	0	0	0	0	1
Calcium	1	1	1	1	1
Phosphorus	1	1	1	1	1
Calculated chemical composition (%)					
Crude protein (%)	13	15	16	13	15.3
Curde fat (%)	2	2	2.5	2.8	4
Crude fiber (%)	15	15	15	10	10
Curde ash (%)	10.5	10	12	10	8
P (%)	1.5	1.5	1.5	0.5	0.8
Ca (%)	0.85	0.5	0.8	1.5	0.7

1) 1 means added, 0 means not added in the formular feed.

**Table 3:** Relative abundance of phyla from various sample type

Phylum (%)	Cattle		Horse	
	
	Feces (n = 5)	Dust (n = 4)	Feces (n = 4)	Dust (n = 4)
Proteobacteria	37.146±11.461	39.844±16.645	42.846±9.128	41.832±19.665
Firmicutes	24.821±13.042	24.959±6.17	21.624±6.163	19.169±8.199
Bacteroidetes	22.162±5.874	10.668±1.989	19.191±5.316	15.483±4.786
Actinobacteria	6.132±2.941	18.644±8.793	9.243±8.895	20.454±11.427
[Thermi]	4.449±2.31	1.646±1.028	0.815±1.509	0.959±0.872
Gemmatimonadetes	0.93±0.469	0.393±0.389	0.058±0.096	0.055±0.031
Tenericutes	0.897±0.443	0.142±0.114	0.206±0.071	0.174±0.125
Chloroflexi	0.893±0.411	1.656±2.064	0.164±0.303	0.215±0.18
Verrucomicrobia	0.594±0.165	0.248±0.164	4.098±3.433	0.674±0.277
Cyanobacteria	0.479±0.187	0.835±0.676	0.114±0.018	0.117±0.026
Planctomycetes	0.451±0.262	0.275±0.194	0.166±0.216	0.198±0.097
Spirochaetes	0.41±0.44	0.067±0.077	0.648±0.458	0.163±0.106
Fibrobacteres	0.179±0.135	0.187±0.19	0.448±0.376	0.071±0.031
Euryarchaeota	0.149±0.087	0.151±0.09	0.079±0.068	0.116±0.106
OP9	0.054±0.077	0.015±0.014	0.001±0.001	0.001±0.002
Synergistetes	0.05±0.057	0.022±0.029	0.046±0.037	0.002±0.003
Acidobacteria	0.048±0.018	0.113±0.083	0.006±0.012	0.005±0.004
TM7	0.037±0.018	0.063±0.032	0.159±0.172	0.163±0.125
BRC1	0.025±0.024	0.021±0.021	0.009±0.009	0.091±0.091
Lentisphaerae	0.022±0.013	0.008±0.006	0.044±0.057	0.005±0.009
WPS-2	0.018±0.018	0.007±0.005	0.01±0.011	0.024±0.013
Fusobacteria	0.013±0.008	0.012±0.005	0.007±0.005	0.012±0.008
NKB19	0.011±0.007	0.005±0.005	0.002±0.004	0.006±0.009
Elusimicrobia	0.01±0.018	0.001±0.001	0.001±0.001	0.001±0.002
WS1	0.003±0.005	0.005±0.005	0±0	0±0
GN02	0.003±0.006	0.001±0.001	0±0	0.001±0.002
LD1	0.002±0.004	0±0	0.002±0.002	0.001±0.001
WWE1	0.002±0.003	0.001±0.001	0±0	0±0
Crenarchaeota	0.002±0.003	0.002±0.001	0±0.001	0±0
Chlorobi	0.001±0.002	0.001±0.001	0±0	0±0
FBP	0.001±0.002	0.001±0.001	0.002±0.004	0.004±0.004
Nitrospirae	0.001±0.002	0.004±0.007	0±0	0±0
Chrysiogenetes	0.001±0.002	0.001±0.001	0±0	0±0
Chlamydiae	0.001±0.001	0±0	0±0	0±0
SAR406	0.001±0.001	0±0	0±0	0±0
Armatimonadetes	0.001±0.001	0.004±0.004	0.001±0.001	0±0
OD1	0±0.001	0±0	0±0	0±0
Thermotogae	0±0.001	0±0	0±0	0±0
SR1	0±0	0.001±0.002	0.012±0.021	0.003±0.004
TM6	0±0	0.001±0.001	0±0.001	0.003±0.004
AD3	0±0	0±0.001	0±0	0±0

Values are presented as mean±standard deviation.

Bacterial candidate division: OP9, TM7, BRC1, WPS-2, NKB19, WS1, GN02, LD1, WWE1, FBP, OD1, SR1, TM6, AD3.

**Table 4:** Relative abundance of genera from various sample type

Genus (%)	Cattle		Horse	
	
	Feces (n = 5)	Dust (n = 4)	Feces (n = 4)	Dust (n = 4)
B-42	4.449±2.31	1.634±1.029	0.815±1.509	0.958±0.872
*Lysobacter*	2.346±1.25	1.35±0.904	0.378±0.679	1.3±0.817
*Luteimonas*	1.994±0.869	2.043±0.952	0.584±0.867	2.226±0.692
*Pseudomonas*	1.823±0.989	3.138±3.577	2.93±3.1	6.424±2.015
*Corynebacterium*	1.628±0.862	3.535±2.517	1.771±0.785	2.201±1.295
*Halomonas*	1.218±0.278	0.435±0.237	0.223±0.427	0.369±0.343
*Staphylococcus*	1.072±0.347	1.693±0.347	0.857±0.32	1.099±0.356
*Clostridium*	1.027±0.46	1.189±0.858	0.291±0.094	0.346±0.141
*Pseudofulvimonas*	0.981±0.787	0.268±0.23	0.058±0.109	0.094±0.077
*Devosia*	0.967±0.403	1.323±0.782	0.501±0.809	1.395±0.604
SMB53	0.908±0.138	0.913±0.385	0.22±0.134	0.363±0.092
*Aequorivita*	0.718±0.944	0.093±0.069	0.238±0.443	0.238±0.243
*Bacillus*	0.542±0.592	1.431±0.353	0.833±0.414	0.786±0.259
*Brumimicrobium*	0.517±0.355	0.12±0.193	0.05±0.071	0.076±0.061
*Acinetobacter*	0.489±0.355	7.853±8.362	27.602±21.758	6.498±2.76
*Janibacter*	0.474±0.486	1.806±1.458	0.273±0.511	0.574±0.427
*Lactobacillus*	0.426±0.138	0.602±0.237	1.703±1.242	1.011±0.224
*Serpens*	0.414±0.37	0.459±0.348	0.308±0.52	0.539±0.804
*Lutibacterium*	0.389±0.113	0.409±0.195	0.254±0.383	0.555±0.228
*Treponema*	0.385±0.415	0.057±0.067	0.638±0.455	0.163±0.106
*Brevibacterium*	0.36±0.131	0.896±0.459	0.642±0.717	2.064±1.365
*Arthrobacter*	0.272±0.221	1.08±1.189	0.575±0.411	2.457±1.937
*Enterococcus*	0.269±0.084	0.408±0.125	0.261±0.143	0.233±0.097
*Cellvibrio*	0.207±0.368	3.299±5.969	0.42±0.686	1.334±0.853
*Sporosarcina*	0.186±0.308	0.966±1.207	0.268±0.353	0.355±0.24
*Jeotgalicoccus*	0.154±0.093	0.478±0.293	0.186±0.284	1.585±1.439
*Methylophaga*	0.147±0.101	0.091±0.054	0.119±0.19	0.35±0.248
*Dietzia*	0.134±0.065	0.678±0.529	0.74±0.557	1.62±1.068
*Stenotrophomonas*	0.129±0.26	0.326±0.464	0.982±1.429	0.507±0.306
*Leucobacter*	0.093±0.063	0.438±0.483	0.162±0.229	0.54±0.317
*Georgenia*	0.083±0.061	0.349±0.093	0.063±0.105	0.17±0.128
*Yaniella*	0.082±0.091	0.529±0.326	0.309±0.548	1.769±1.298
*Agrobacterium*	0.052±0.098	0.229±0.259	0.351±0.363	0.872±1.314
*Ruminococcus*	0.397±0.427	0.118±0.053	0.222±0.135	0.044±0.026
*Prevotella*	0.079±0.11	0.024±0.032	0.307±0.446	0.017±0.01
Others	74.589±3.7	59.741±12.006	53.866±12.051	58.865±5.98

Values are presented as mean±standard deviation.

**Table 5:** List of genus significantly different between the sample types

Groups	Genus	Relative abundnce (%)				p-value**

		C_F (%)	C_D (%)	H_F (%)	H_D (%)	
C_F and C_D	*Halomonas*	**1.22**	0.44	-	-	0
	*Georgenia*	0.08	**0.35**	-	-	0
	*Bacillus*	0.54	**1.43**	-	-	0.03
	*Staphylococcus*	1.07	**1.69**	-	-	0.03
C_F and H_F	*SMB53*	**0.91**	-	0.22	-	0
	*Halomonas*	**1.22**	-	0.22	-	0.01
	*Clostridium*	**1.03**	-	0.29	-	0.02
	*Lysobacter*	**2.35**	-	0.38	-	0.02
	*B-42*	**4.45**	-	0.81	-	0.03
	*Streptococcus*	0.15	-	**3.44**	-	0.03
	*Brumimicrobium*	**0.52**	-	0.05	-	0.04
	*Luteimonas*	**1.99**	-	0.58	-	0.05
C_D and H_D	*Streptococcus*	-	0.19	-	**3.23**	0.02
	*Bacillus*	-	**1.43**	-	0.79	0.03
	*Lysinibacillus*	-	**0.56**	-	0.12	0.04
	*HTCC*	-	0.08	-	**0.32**	0.05
	*Lactobacillus*	-	0.6	-	**1.01**	0.05
H_F and H_D	*Luteimonas*	-	-	0.58	**2.23**	0.03

Bold number means the higher abundance ratio between groups.

** The p values were determined using student’s t test.

**Table 6:** The overview of classfied sequencing reads from whole genome sequencing data

Items	Cattle_feces (n = 5)	Cattle_dust (n = 4)	Horse_feces (n = 4)	Horse_dust (n = 4)
No. of average filtered reads1)	221,580±71,412	825,577±690,824	313,191±58,230	608,407±265,166
Bacteria2)	218,651±70,808	819,677±689,692	306,884±56,758	605,846±264,408
Bacteria (%)	98.6	99.0	98.0	99.6
Archaea2)	2,158±910	1,395±1,094	4,076±1,699	550±332
Archaea (%)	1.0	0.3	1.4	0.1
Viruses2)	336±308	2,414±2,099	1,737±1,543	1,173±566
Viruses (%)	0.1	0.3	0.5	0.2
Unclassified2)	433±189	2,090±2,500	492±119	836±371
Unclassified (%)	0.2	0.3	0.2	0.1

1) Reads number passing the in-house perl scripts and Cutadapt 1.14.

2) Percentage of reads classified (over 97% identity) towards RefSeq sequences included in Kraken database.

**Table 7:** Aligned virus read percentage (%) in order level

Domain	Cattle_feces	Cattle_dust	Horse_feces	Horse_dust
*Caudovirales*	49.5	75.7	93.5	89.2
*Herpesvirales*	0.2	0.1	0.2	0.1
*Bunyavirales*	0.0	0.0	0.0	0.0
*Nidovirales*	0.0	0.3	0.1	0.2
*Picornavirales*1)	0.0	0.0	0.0	0.0
Others2)	50.3	24.0	6.3	10.5
Total	100.0	100.0	100.0	100.0

1) Foot-and-mouth disease (FMD) is a virus of the family *Picronavirales*.

2) Including unclassified and not taxonomic classified in order level.

**Table 8:** Relative abundance of virus from various sample type in genus level

Phage	Cattle		Horse	
	
	Feces	Dust	Feces	Dust
*Mycobacterium*	2.13±3.03	0.28±0.15	15.66±16.91	4.32±2.59
*Pseudomonas*	4.78±1.43	9.2±6.81	21.95±20.31	6.77±3.63
*Streptococcus*	0.19±0.42	0.04±0.05	4.69±2.27	0.68±0.13
*Escherichia*	4.52±5.51	3.98±2.2	8.23±6.05	41.38±17.41
*Xylella*	2.59±2.93	0.06±0.1	0±0	0.5±0.93
*Aureococcus*	0.73±0.64	0±0	0.03±0.06	0±0
*Cronobacter*	0.96±0.95	3.27±1.93	0.15±0.17	1.11±0.5
*Morganella*	0.19±0.31	0.28±0.55	0±0	0±0
*gemycircularvirus*	14.47±21.21	14.44±20.38	0.35±0.66	0.39±0.42
*Salmonella*	0.57±0.93	7.82±14.58	1.57±1.56	1.32±0.77
*Bacillus*	0.14±0.32	0.67±1.03	0.41±0.8	0.27±0.11
*Staphylococcus*	0.62±1.38	2.64±2.18	0.15±0.18	2.01±0.98
*Erwinia*	0.19±0.31	3.32±3.62	0.48±0.56	3.72±7.15
*Rhodococcus*	0.79±1.23	0.23±0.34	5.75±6.23	0.31±0.34
*Aeromonas*	3.49±3.66	0.78±0.97	0.33±0.35	0±0
*Ailuropoda*	0.79±0.76	0.69±0.86	0.95±1.32	0.54±0.48
*Enterobacteria*	0.43±0.95	0.49±0.24	0.74±1.12	0.99±0.46
*Klebsiella*	1.31±2.56	0.38±0.11	0.17±0.29	0.37±0.33
*Gordonia*	2.31±3.2	8.32±16.02	3.17±1.28	1.65±1.01
*Clostridium*	0.8±1.03	0±0	0±0	0±0
*Enterococcus*	2.85±4.56	1.7±1.39	0.77±1.13	0.25±0.14
Others	55.15±42.68	41.41±26.49	34.45±38.75	33.42±62.62

Values are presented as mean±standard deviation.

## References

[1] Tullo E, Finzi A, Guarino M (2019). Review: Environmental impact of livestock farming and precision livestock farming as a mitigation strategy. Sci Total Environ.

[2] Kang HJ, Piao MY, Park SJ, Na SW, Kim HJ, Baik M (2019). Effects of ambient temperature and rumen-protected fat supplementation on growth performance, rumen fermentation and blood parameters during cold season in korean cattle steers. Asian-Australas J Anim Sci.

[3] Woo JH, Chae HS, Kim NY (2017). Effect of concentrate feed level on weight change, intestinal microbiota, and blood profiles of jeju cross-bred horses. Ann Anim Resour Sci.

[4] Grubman MJ, Baxt B (2004). Foot-and-mouth disease. Clin Microbiol Rev.

[5] Morrison PK, Newbold CJ, Jones E (2018). The equine gastrointestinal microbiome: impacts of age and obesity. Front Microbiol.

[6] Han GG, Lee JY, Jin GD (2018). Tracing of the fecal microbiota of commercial pigs at five growth stages from birth to shipment. Sci Rep.

[7] Lee M, Jeong S, Seo J, Seo S (2019). Changes in the ruminal fermentation and bacterial community structure by a sudden change to a high-concentrate diet in korean domestic ruminants. Asian-Australas J Anim Sci.

[8] Kraemer JG, Ramette A, Aebi S, Oppliger A, Hilty M (2018). Influence of pig farming on the human nasal microbiota: Key role of airborne microbial communities. Appl Environ Microbiol.

[9] Timsit E, Workentine M, van der Meer F, Alexander T (2018). Distinct bacterial metacommunities inhabit the upper and lower respiratory tracts of healthy feedlot cattle and those diagnosed with bronchopneumonia. Vet Microbiol.

[10] Leclere M, Lavoie-Lamoureux A, Lavoie JP (2011). Heaves, an asthma-like disease of horses. Respirology.

[11] McKenzie VJ, Song SJ, Delsuc F (2017). The effects of captivity on the mammalian gut microbiome. Integr Comp Biol.

[12] Budiansky S (1997). The nature of horses.

[13] Hall JB (2001). Nutrition and feeding of the cow-calf herd: Digestive system of the cow.

[14] Apajalahti J (2005). Comparative gut microflora, metabolic challenges, and potential opportunities. J Appl Poult Res.

[15] O’Donnell MM, Harris HMB, Ross RP, O’Toole PW (2017). Core fecal microbiota of domesticated herbivorous ruminant, hindgut fermenters, and monogastric animals. Microbiologyopen.

[16] Henderson G, Cox F, Ganesh S (2015). Rumen microbial community composition varies with diet and host, but a core microbiome is found across a wide geographical range. Sci Rep.

[17] He J, Sakaguchi K, Suzuki T (2012). Acquired tolerance to oxidative stress in *Bifidobacterium longum* 105-A via expression of a catalase gene. Appl Environ Microbiol.

[18] Li J, Paredes-Sabja D, Sarker MR, McClane BA (2016). *Clostridium perfringens* sporulation and sporulation-associated toxin production. Microbiol Spectr.

[19] Ventura M, Canchaya C, Tauch A (2007). Genomics of *Actinobacteria*: Tracing the evolutionary history of an ancient phylum. Microbiol Mol Biol Rev.

[20] Gomes KM, Duarte RS, de Freire Bastos, do Carmo Maria (2017). Lantibiotics produced by *Actinobacteria* and their potential applications (a review). Microbiology.

[21] Myer PR, Smith TPL, Wells JE, Kuehn LA, Freetly HC (2015). Rumen microbiome from steers differing in feed efficiency. Plos One.

[22] Liu C, Meng Q, Chen Y (2017). Role of age-related shifts in rumen bacteria and methanogens in methane production in cattle. Front Microbiol.

[23] Wang X, Yang H, Zhang Y (2015). *Luteimonas soli* sp. nov., isolated from farmland soil. Int J Syst Evol Microbiol.

[24] Venable E, Bland S, McPherson J, Francis J (2016). Role of the gut microbiota in equine health and disease. Anim Front.

[25] Hardie J, Whiley R (1995). The genus streptococcus, in the genera of lactic acid bacteria.

[26] Boyle A, Timoney JF, Newton J, Hines M, Waller A, Buchanan B (2018). *Streptococcus equi* infections in horses: Guidelines for treatment, control, and prevention of strangles—revised consensus statement. J Vet Intern Med.

[27] Chen L, Zhang Y, Wang S, Zhang Y, Huang T, Cai Y (2017). Prediction and analysis of essential genes using the enrichments of gene ontology and KEGG pathways. Plos One.

[28] Uhlik O, Wald J, Strejcek M (2012). Identification of bacteria utilizing biphenyl, benzoate, and naphthalene in long-term contaminated soil. Plos One.

[29] Ackermann HW (1998). Tailed bacteriophages: the order caudovirales. Adv Virus Res.

[30] Park EJ, Kim KH, Abell GCJ, Kim MS, Roh SW, Bae JW (2011). Metagenomic analysis of the viral communities in fermented foods. Appl Environ Microbiol.

