# Supplementary material for: Differences in microbiome and virome between cattle and horses in the same farm
Source: Asian-Australas J Anim Sci. 2019 Oct 22;33(6):1042–55. doi: 10.5713/ajas.19.0267 (PMC7206377; doi:10.5713/ajas.19.0267)
Supplement: Supplementary file 1 [file ajas-19-0267-suppl.pdf]

Supplementary Table 1. Information of individual cattle and horses in each sampling site.

Individual no.	Species	Sex	Month of age	Stage	Feedlot
1	Hanwoo	Female	92.2	Fattening	C
2	Hanwoo	Female	52.0	Fattening	C
3	Hanwoo	Female	49.6	Pregnant	C
4	Hanwoo	Female	49.6	Pregnant	C
5	Hanwoo	Female	49.6	Pregnant	C
6	Hanwoo	Female	44.0	Pregnant	C
7	Hanwoo	Female	38.4	Pregnant	C
8	Hanwoo	Female	37.4	Pregnant	C
9	Hanwoo	Female	31.7	Pregnant	C
10	Hanwoo	Female	31.3	Pregnant	C
11	Hanwoo	Female	31.0	Pregnant	C
12	Hanwoo	Female	30.9	Pregnant	C
13	Hanwoo	Female	30.5	Pregnant	C
14	Hanwoo	Female	24.0	Breeding	C
15	Hanwoo	Female	19.3	Breeding	C
16	Hanwoo	Female	19.2	Breeding	C
17	Hanwoo	Female	19.2	Breeding	C
18	Hanwoo	Female	19.0	Breeding	C
19	Hanwoo	Female	18.6	Breeding	C
20	Hanwoo	Female	16.6	Breeding	C
21	Hanwoo	Female	14.6	Breeding	C
22	Hanwoo	Female	14.4	Breeding	C
23	Hanwoo	Female	14.3	Breeding	C
24	Hanwoo	Female	13.6	Breeding	C
25	Hanwoo	Female	13.6	Breeding	C
26	Hanwoo	Female	12.7	Breeding	C
27	Hanwoo	Female	4.2	Weaning	C
28	Hanwoo	Male	3.4	Weaning	C
29	Hanwoo	Female	3.0	Weaning	C
30	Hanwoo	Female	0.9	Suckling	C
31	Hanwoo	Female	0.7	Suckling	C
32	Hanwoo	Male	0.2	Suckling	C
33	Hanwoo	Male	0.1	Suckling	C
34	Hanwoo	Male	18.8	Fattening	A
35	Hanwoo	Male	18.4	Fattening	A
36	Hanwoo	Male	14.6	Fattening	A
37	Hanwoo	Male	14.5	Fattening	A
38	Hanwoo	Male	14.1	Fattening	A
39	Hanwoo	Male	13.8	Fattening	A
40	Hanwoo	Male	13.6	Fattening	A
41	Hanwoo	Male	13.3	Fattening	A

42	Hanwoo	Male	11.9	Fattening	A
43	Hanwoo	Male	11.1	Fattening	A
44	Hanwoo	Male	6.8	Weaning	A
45	Hanwoo	Male	5.8	Weaning	A
46	Hanwoo	Male	5.8	Weaning	A
47	Hanwoo	Male	5.7	Weaning	A
48	Hanwoo	Male	5.7	Weaning	A
49	Hanwoo	Male	5.4	Weaning	A
50	Hanwoo	Male	4.0	Weaning	A
51	Hanwoo	Male	2.4	Weaning	A
52	Hanwoo	Male	1.7	Weaning	A
53	Hanwoo	Male	1.6	Weaning	A
54	Hanwoo	Male	0.5	Suckling	A
55	Holestein	Female	52.5	Dairy_Pregnant	B-2
56	Holestein	Female	37.5	Dairy_Pregnant	B-2
57	Holestein	Female	29.9	Dairy_Pregnant	B-2
58	Holestein	Female	19.4	Dairy_Breeding	B-2
59	Hanwoo	Female	14.7	Breeding	B-1
60	Hanwoo	Female	12.4	Breeding	B-1
61	Hanwoo	Female	12.3	Breeding	B-1
62	Holestein	Female	11.5	Dairy_Breeding	B-2
63	Holestein	Female	11.3	Dairy_Breeding	B-2
64	Hanwoo	Female	6.8	Weaning	B-1
65	Hanwoo	Female	6.0	Weaning	B-1
66	Hanwoo	Female	5.9	Weaning	B-1
67	Hanwoo	Female	5.8	Weaning	B-1
68	Hanwoo	Female	5.8	Weaning	B-1
69	Hanwoo	Female	5.4	Weaning	B-1
70	Hanwoo	Female	4.5	Weaning	B-1
71	Hanwoo	Female	1.1	Suckling	B-1
72	Throughbred	Female	139.0	Fattening	D
73	Hallla	Female	16.2	Weaning	D
74	Hallla	Female	113.9	Pregnant	D
75	Hallla	Female	38.7	Breeding	D
